# Before a positive effect of classical music on chronic neck pain can be demonstrated, all potential interfering factors must be eliminated

**DOI:** 10.1590/1806-9282.20252305

**Published:** 2026-08-21

**Authors:** Carla Alexandra Scorza, Fulvio Alexandre Scorza, Josef Finsterer

**Affiliations:** 1 Universidade Federal de São Paulo, Escola Paulista de Medicina – São Paulo (SP), Brazil.; 2 Neurology and Neurophysiology Center, Department of Neurology – Vienna, Austria.

Dear Editor,

We read with interest the article by Torlak et al. on the effect of classical music in combination with physical therapy over a period of 4 weeks on pain, anxiety, and quality of life (QoL) in 20 patients with chronic neck pain, measured using the visual analog scale (VAS), the Beck Anxiety Inventory (BAI), the Neck Disability Index (NDI), and SF-36 physical health scores^1^. The control group included 20 patients with neck pain who received only physical therapy^1^. After treatment, VAS scores, NDI, and BAI decreased, while SF-36 scores increased in both the treatment group and the control group^1^. The VAS scores decreased more in the treatment group than in the control group^1^. The study is interesting, but some points should be discussed.

The first point is that it was not reported whether all 40 patients with chronic neck pain also suffered from anxiety and a deterioration in their quality of life^1^. Since anxiety and a deterioration in QoL may not be present in every patient with chronic neck pain, we should know whether all patients really suffered from anxiety and a deterioration in their QoL in addition to pain. How many patients had normal BAI and NDI scores before treatment?

The second point is that the effect of music therapy depends heavily on the cause of the neck pain. Since neck pain can have many causes, the effect of music can vary greatly depending on the underlying cause of the neck pain. Causes of chronic neck pain can include muscle tension due to a hunched posture in front of a computer or smartphone, reading in bed, poor sleep quality, psychiatric disorders, personality type, internal or external stress, a history of whiplash, sports injuries, falls, heavy lifting, osteoarthritis, disc disorders without impact on the nerve roots, bone spurs, rheumatoid arthritis, aseptic meningitis, or malignant tumors. What were the causes of chronic neck pain in the patients included in the study, and did the treatment have different effects on neck pain depending on the underlying cause?

The third point is that the effect of music can also depend heavily on the type and quality of the music. Did all 20 patients really like classical music? Those who prefer jazz, soul, pop, rock, hip-hop, electronic music, country, rhythm and blues, bossa nova, or world music may find classical music more disturbing than relaxing, anxiety-relieving, or antidepressant.

The fourth point is that the number of patients in the treatment group who consumed alcohol and were married was higher than in the control group^1^. Therefore, the reduction in VAS in the treatment group could simply be due to regular alcohol consumption and regular sexual activity in the treatment group. These confounding variables should be excluded.

The fifth point is that the effect of any intervention, including music for chronic neck pain, may also depend on comorbidities and concomitant medications, as well as regular consumption of adrenergic stimulants (coffee, tea, cola, and Red Bull) or illegal drugs. Although the patients included were relatively young, it cannot be ruled out that these factors had an influence on the effect of the treatment, which is why these confounding factors should be excluded from the analysis.

The sixth point is that the sample size of 20 patients is too small to draw general conclusions. Larger groups should be studied to assess whether classical music is truly beneficial for patients with chronic neck pain in addition to physical therapy.

Overall, before attributing a positive effect to classical music on chronic neck pain, larger groups should be studied and analyzed that are homogeneous in terms of the causes of neck pain, music preferences, type of work, hobbies, diet, drinking habits, comorbidities, and concomitant medication. Confounding variables must be excluded before it can be concluded that Pachelbel's music is beneficial for the treatment of neck pain.

## Data Availability

The datasets generated and/or analyzed during the current study are available from the corresponding author upon reasonable request.
